# Preclinical Pharmacokinetics, Biodistribution, and Acute Toxicity Evaluation of Caerin 1.9 Peptide in Sprague Dawley Rats

**DOI:** 10.1155/2022/9869293

**Published:** 2022-08-02

**Authors:** Xiaodan Yang, Junjie Li, Shu Chen, Liyin Xiao, Dongmin Cao, Xiaolian Wu, Hejie Li, Guoying Ni, Tianfang Wang, Guoqiang Chen, Xiaosong Liu

**Affiliations:** ^1^The First Affiliated Hospital of Guangdong Pharmaceutical University, Guangzhou, Guangdong 510080, China; ^2^Cancer Research Institute, First People's Hospital of Foshan, Foshan, Guangdong 528000, China; ^3^Genecology Research Centre, University of the Sunshine Coast, Maroochydore DC, QLD 4558, Australia; ^4^Department of Rheumatology, First People's Hospital of Foshan, Foshan, Guangdong 528000, China

## Abstract

Caerin 1.9 is a natural peptide derived from the skin secretions of the Australian tree frog (*Litoria*) with broad-spectrum antimicrobial and anticancer bioactivity. It improves the efficacy of a therapeutic vaccine and immune checkpoint inhibitor therapy when injected intratumorally and inhibits TC-1 tumor growth when applied topically through intact skin in a TC-1 murine tumor model. This paper investigated the pharmaceutical kinetic profile, the tissue distribution, and the acute safety investigation of Caerin 1.9 peptide in Sprague Dawley (SD) rats. The results showed that subcutaneous injection of Caerin 1.9 at 100 mg/kg is safe and does not cause mortality or organ malfunction in the recipient rats. For the consecutive injection of F3 at 10 mg/kg, the peak concentration (*C*_max_) of F3 displayed at 1 hr after injection in male rats was 591 ng/mL, the average drug retention time was 0.807 hr, *T*_1/2_ was 4.58 hr, and AUC_0-last_ was 1890 h × ng/mL. In female rats, *C*_max_ was 256 ng/mL, with an average drug retention time of 2.96 hr, *T*_1/2_ of 1.33 hr, and AUC_0-last_ of 740 h × ng/mL. The results showed that the concentration of Caerin 1.9 in the peripheral blood peaked at 1 hour. As injected concentration increased, *T*_1/2_ extended, and *C*_max_, AUC_0-last_, and volume of distribution at a steady state all increased. After 14 days of repeated subcutaneous injection at 10.0 mg/kg, no accumulation of Caerin 1.9 in plasma was observed. The results of tissue distribution showed that the Caerin 1.9 is below the LC-MS/MS detection threshold at a minimum concentration of 40 ng/g. In conclusion, Caerin 1.9 is well tolerated in rats and could be used with current immunotherapies for better management of solid tumors and genital warts.

## 1. Introduction

The human papillomavirus (HPV) is a DNA virus that transmits from person to person mainly through sexual contact. There are more than 200 subtypes of HPV. HPV is categorized into high-risk and low-risk subtypes based on their carcinogenic potential. A variety of HPV-related malignant tumors are linked to more than 12 subtypes, including high-risk HPV: HPV16, 18, 31, 33, 35, 39, 45, 51, 52, 56, 58, and 59 [1–4].

Cervical cancer is one of the most common gynecological tumors in women worldwide, especially in developing countries where HPV prophylactic vaccines are less widely available [5]. The main capsid protein L1, which can self-assemble into forming virus-like particles (VLPs), is used to create HPV prophylactic vaccines. The vaccinations efficiently prevent cervical cancer and may ultimately lower its incidence rate. However, the HPV prophylactic vaccines do not have therapeutic effects on infected individuals. Therefore, better HPV treatments, particularly effective therapeutic vaccinations against HPV-related malignancy, are urgently needed.

Natural peptides have been attracting more attention for their potential therapeutical significance in recent years. A number of peptide-based medications have been developed, such as adrenocorticotrophic hormone isolated from pituitary glands of bovine and pig for the treatment of acute gout [6–8], as well as calcitonin isolated from the parotid gland of salmon to reduce calcium migration from bone to blood [9]. Natural peptides have advantages over protein and other small molecule-based medications, including low cost, ease of production, high purity, few side effects, and good buffer compatibility [8, 10, 11].

Caerin peptides are a family of host defense peptides identified from the skin secretion of the Australian tree frog (*Litoria* genus), which have a broad spectrum of antimicrobial and antitumor properties [12]. Among them, Caerin 1 inhibits HIV transmission *in vitro* at a low concentration of 6.25–25 *µ*M [13], and previous studies have shown that Caerin 1.1 has a profound antitumor impact on a variety of tumor cells *in vitro* [9]. Caerin 1.9 has an antibacterial effect and outperforms polymyxin B when it comes to suppressing standard *S. aureus*, MRSA, *A. baumannii*, and *S. haemolyticus*. Caerin 1.9 exhibits effective antibacterial activity without inducing resistance after 30 passages, and Caerin peptides in a temperature-sensitive gel formulation inhibited bacteria growth in the skin of MRSA-infected mice [14, 15]. Another study found that its immobility property improves metal corrosion resistance significantly [16]. Caerin 1.1 and 1.9 inhibit the growth of a variety of cancer cells *in vitro*, including MCF-7 and SKBR-3 breast cancer cells, B-CPAP and CAL-62 thyroid cancer cells, and HPV+ cell lines TC-1 and Hela cells [17–19]. Proteomic analysis of HeLa cells treated with Caerin 1suggested that Caerin 1.1 and 1.9 inhibited the proliferation of HeLa cells *in vitro* via the TNF-*α* signaling pathway [17]. Caerin 1.1 and 1.9 also inhibit TC-1 cell growth in mice, but the inhibition requires the existence of an intact adaptive immune system, as the effect disappeared in nude mice lacking B and T cells [3, 9]. The combined use of Caerin 1.1 and 1.9 with a therapeutic vaccine boosts the efficacy of the HPV16 E7 peptide-based therapeutic vaccine by recruiting more T cells to tumor sites [12]. Moreover, in mice, transdermal administration of Caerin 1.1 and 1.9 in a temperature-sensitive gel suppresses the formation of subcutaneously implanted TC-1 tumors [20]. Therefore, Caerin 1.1 and Caerin 1.9 are candidates for clinical development as antitumor or antimicrobial therapies.

Caerin 1.1 and Caerin 1.9 are stable. They remained active following incubation at room temperature for 7 days in a low pH environment (pH5.5-7), and, when formed in a temperature-sensitive gel, they maintained a similar degree of bioactivity after incubation at room temperature for 30 days. Furthermore, after being heated at 100°C for 5 minutes, they are equally effective in preventing the development of TC-1 cells [12, 20].

The acute toxicity, pharmacokinetics, and tissue distribution of Caerin 1.9 in SD rats were addressed in this work as a foundation for further exploring the feasibility of developing Caerin 1 peptide-based therapies.

## 2. Materials and Methods

### 2.1. Details of Experimental Compounds

Caerin 1.9 (GLFGVLGSIAKHVLPHVVPVIAEKL-NH_2_), simplified as F3, was synthesized by Mimotopes Pharmaceutical Limited in Wuxi, China. The purity of F3 was more than 99.36% by Mimotopes RP-HPLC, and it was stored at −20°C a until usage. 0.9% sodium chloride injection (batch number: F18081407) purchased from Qingzhou Yaowang Pharmaceutical Co., Ltd. was used for dilution and delivery media. Protease inhibitors (batch number: SLBZ8644) and 10% neutral formalin buffer were provided by Sigma-Aldrich Trading Co., Ltd. (Shanghai).

### 2.2. Rats

Sprague Dawley rats (CRL: CD (SD) IGS (SPF/VAF)) were obtained from Beijing Weitong Lihua Experimental Animal Technology Co., Ltd. The rats were kept at a specific pathogen free (SPF) facility on a 12 hr light/dark cycle at 22°C with a humidity of 75%, and they were provided with disinfected standard food and water. On the day of the experiment, the male rats weighed 230–290 g and the female rats weighed 180–240 g. They were aged 7–9 weeks.

**Figure 1:**
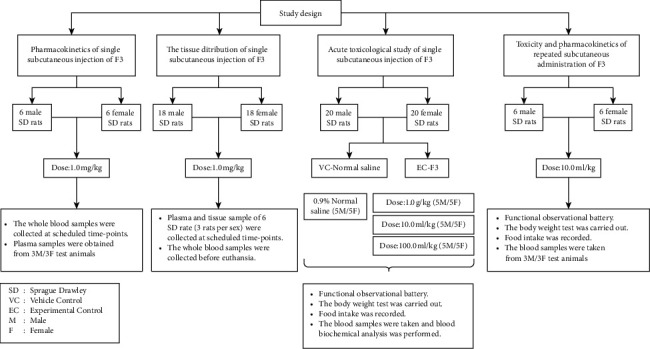
Schematic flowchart of study design.

The animal experiments were approved by the animal ethics committee of Foshan First People's Hospital (no. FAHGPU20160316) and Kang Long Hua Cheng Co., Ltd, where all experiments were performed (no. 20-012).  Figure 1 summarizes the experimental processes, which evaluated the pharmacokinetics, toxicity, and biodistribution of a single subcutaneous dose [21], as well as the toxicity and pharmacokinetics of repeated subcutaneous administration of F3.

### 2.3. Functional Observation

The mortality, general health status, and toxic reaction symptoms of the experimental groups were documented. Before the first day of F3 administration and during the experiments, a detailed clinical observation was carried out and recorded the changes in skin, eyes and mucous membrane, respiratory system, nervous system, activity, and behaviors of the rats. The body weight was measured and recorded before the first day of administration.

### 2.4. Pharmacokinetics of Single and Repeated Subcutaneous Injection of F3

A single subcutaneous injection of 1.0 mg/kg F3 was given to a total of 12 SD rats (6 females, 6 males). Before and after F3 administration, whole blood samples were collected at predetermined time intervals (0, 0.5, 1, 2, 4, 6, 8, 24, 48, 72, 96, 144, and 192 hr). At each time point, at least 0.5 ml blood samples were taken alternatively from 3M/3F rats.

A total of 12 SD rats (6 males, 6 females) received a daily subcutaneous injection of F3 at a dose of 10.0 mg/kg for 14 days. The first day of F3 administration was recorded as day 1. At least 0.5 ml blood samples were obtained alternatively from 3M/3F rats at pre- and post-F3 injection time periods (0, 0.5, 1, 2, 4, 6, 8, and 24 hr).

### 2.5. Pharmacokinetic Analysis

The concentration of F3 in rat plasma was determined by liquid chromatography-tandem mass spectrometry, using the method established by Kanglong Huacheng Co., Ltd. The analyte was assayed by liquid chromatography coupled with the tandem mass spectrometry (LC-MS/MS) method. The LC-MS/MS system consisted of a Shimadzu HPLC system (Kyoto, Japan) and an LCMS-8060 instrument (serial no. 011105500332 AE). The following were the LC conditions: column, HALO ES-C18 2.7 *µ* C18 160A (50 × 2.1 mm); gradient elution at 0.5 mL/min with 5% acetonitrile in water containing 0.1% formic acid or 95% acetonitrile in water containing 0.1% formic acid; and injection volume of 10 *µ*L. A multiple reaction monitoring (MRM) method was selected for quantitative analysis. For analyte and internal standard, the optimum transitions were 519.50 > 129.15 (five charges) and 649.15 > 120.10 (four charges), 393.20 > 373.25, and 237.15 > 194.15, respectively. The instrumental parameters were set as follows: nebulizing gas flow was 3 L/min; heating gas flow was 10 L/min; interface temperature was 300°C; DL temperature was 250°C; and heat block temperature was 400°C.

PK parameters were estimated by a noncompartmental model with the calculation model of “Linear Trapezoidal Linear Interpolation” using WinNonlin (Phoenix TM, version 8.1). If the plasma F3 was detected, the following pharmacokinetic parameters were measured and calculated: *C*_max_ (maximum blood concentration), *T*_max_ (peak time), *T*_1/2_ (elimination half-life), AUC (area under plasma concentration-time curve), Vz_F_obs (apparent distribution volume), Cl_F_obs (clearance rate), and MRT_Inf__obs (mean residence time).

### 2.6. The Tissue Distribution of Single Subcutaneous Injection of F3

Thirty-six SD rats (18 females, 18 males) were randomly selected and given a single subcutaneous injection of F3 at 1.0 mg/kg. Tissue samples were collected at 1, 2, 4, 8, 24, and 48 hours after F3 administration. At each time point, six rats (3 of each sex) were sacrificed. At least 0.5 ml of whole blood was collected before euthanasia, and 0.5–1.0 g of tissue was collected after euthanasia [22]. The heart, brain, liver, kidney, lung, spleen, stomach, small intestine, testis, ovary, subcutaneous fat, and skeletal muscle were among the tissue types studied. The types of tissues to be collected, collection sites, and relevant precautions are shown in Table 1.

### 2.7. Acute Toxicological Study of Single and Repeated Subcutaneous Injection of F3

Forty SD rats (20 males and 20 females) were randomly divided into 4 groups (Figure 1). All rats were given F3 once on the day of the experiment. The administration doses are listed in Table 2.

Twelve SD rats (6 males, 6 females) were given a daily subcutaneous injection of F3 at a dose of 10.0 mg/kg for consecutive 14 days. The first day of administration was recorded as day 1.

For 14 days, the body weight was measured before F3 administration on the first day of the experiment and once a week thereafter, with additional measurements on the day before and on the day of the planned autopsy. Food intake was recorded once a week. The rats were anesthetized by inhalation of 70% CO_2_/30% O_2_ before dissection, and blood samples were taken for hematology and biochemical analysis. The collected tissue was weighed and preserved for pathological examination.

### 2.8. Hematology Analysis

Blood smears were used to investigate the morphology of basophils (ABBASO), eosinophils (ABEOS), neutrophils (ABNEUT), monocytes (ABMONO), and lymphocytes (ABLYMP) in the peripheral blood. When white blood cell classification and reticulocyte count cannot be calculated directly by ADVIA 2120i, they were manually counted and calculated following the procedure published previously [23]. The number of platelets was determined manually when they could not be counted mechanically because of coagulation. Wright-Giemsa staining was used to prepare blood smears for cell morphology [24].

### 2.9. Blood Biochemical Analysis

Liver and renal functions were measured with special attention to alanine aminotransferase (ALT), aspartate aminotransferase (AST), alkaline phosphatase (ALP), serum creatinine, total bilirubin, and urea levels. In addition, the levels of glucose, calcium, total protein, and albumin in serum samples were measured. The blood biochemical test, hematology test, and coagulation function test were detected by Hitachi 7180 automatic biochemical analyzer, Siemens ADVIA 2120i hematology analyzer, and Stargo Emo Express Hemagglutination analyzer, respectively.

### 2.10. Gross Dissection and Histopathological Examination of Important Tissues

After blood was collected at the predetermined time points, all rats were euthanized and subjected to a comprehensive general autopsy, which included a thorough examination of the external surface of the body, all foramen, cranial cavity, external surface of the brain, chest, abdomen, pelvic cavity, viscera, cervix, and genitalia. The adrenal gland, brain (brain, cerebellum, and brain stem), epididymis, heart, kidney, liver, ovary, spleen, testis, thymus, thyroid gland, and uterus were carefully collected, weighed, and documented before being kept in 10% neutral formalin buffer (10% NBF).

The H&E staining was followed as reported elsewhere [16]. Following formalin fixation, the important tissues were dehydrated, stained with hematoxylin (about 15 minutes) and eosin (1–3 minutes), dehydrated again with gradient alcohol, clarified in xylene, and then examined and recorded under the microscope [25].

### 2.11. Statistical Analysis

The mean and standard deviation (SD) of all variables were calculated. The data of each group was represented by the gender set of all parameters. The differences between groups were tested using univariate analysis of variance (ANOVA) and least significant difference (LSD). If heterogeneity was observed in a set of data, nonparametric Kruskal-Wallis and Mann-Whitney *U* tests were performed to determine the difference of 95% CI (*P* < 0.05). The results were considered significantly credible when *P* < 0.05. The data were analyzed using GraphPad Prism 8.0 software [26].

## 3. Result

### 3.1. Plasma Pharmacokinetics

First, we looked at the pharmacokinetic characteristics of F3 when given subcutaneously at 1.0 mg/kg once or subcutaneously at 10.0 mg/kg for 14 days.

**Figure 2:**
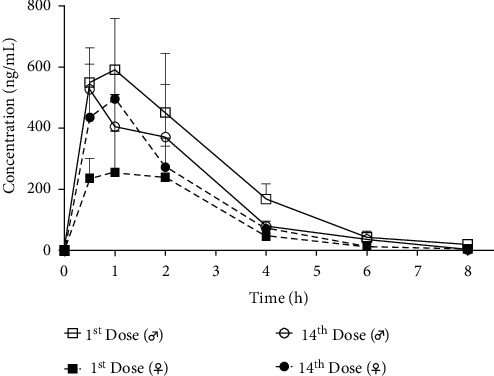
The concentration-time profiles of F3 after a single dose (10.0 mg/kg) and after repeated daily dosing with 10.0 mg/kg of F3 for 14 days. (a) The concentration-time profiles in plasma following subcutaneous injection of F3 at 1.0 mg/kg once. (b) The concentration-time profiles after a single dose (10.0 mg/kg) and repeated daily dosing with 10.0 mg/kg of F3 for 14 days.

Plasma concentrations of F3 were analyzed by HPLC, and the pharmacokinetic parameters are summarized in Table 3. A single subcutaneous injection of F3 (1.0 mg/kg) resulted in a rapid increase of F3 within 1 hour followed by a gradual drop over the next 6 hours (Figure 2). F3 reached its peak concentration in male rats 1 hr after administration, giving *C*_max_ of 256 ng/mL, a mean drug retention time of 2.05 hr, *T*_1/2_ of 1.16 hr, and AUC_0−last_ of 661 h × ng/mL. In female rats, the peak concentration attained 110 ng/mL at 0.5 hr after administration. Female rats had a shorter average retention period of 1.42 hours than male rats and provided *T*_1/2_ of 0.677 hr and AUC_0−last_ of 216 h × ng/mL. Furthermore, the steady-state volumes of distribution of male and female rats were 2431.95 mL/kg and 4402.21 mL/kg, respectively.

For the consecutive injection of F3 at 10 mg/kg, the peak concentration of F3 reached 591 ng/mL at 1 hour after injection in male rats, with an average drug retention time of 0.807 hr, *T*_1/2_ of 4.58 hr, and AUC_0−last_ of 1890 h × ng/mL. The volume of distribution was 34647.55 mL/kg at a steady state. The peak concentration of F3 in female rats also appeared at 1 hour after injection; however, their plasma level was substantially lower than that of males, with *C*_max_ of 256 ng/mL, an average drug retention time of 2.96 hr, *T*_1/2_ of 1.33 hr, and AUC_0−last_ of 740 h × ng/mL. Similarly, the steady-state volume of distribution that was observed in females was lower than that in males, at a volume of 25498.49 mL/kg. The results showed that F3 had a short peak time, indicating that F3 had good absorption, rapid onset, and fast elimination from the peripheral circulation. With the increase of drug concentration, the values of *C*_max,_ AUC_0−last_, and the volume of distribution at a steady state increased simultaneously. As the results of a single treatment, male rats had higher peak concentration and steady-state volume of distribution than female rats.

The ratios of *C*_max_ and AUC_last_ in females and males after repeated injections of F3 were 2.309 and 2.554, respectively, after the first day of administration. On the 14th day, the postinjection ratio was 1.063 in females and 1.152 in males. After first dosing, *C*_max_ and AUC_last_ in male rats were higher than those in female rats, while, after repeated doses, both male and female rats had similar *C*_max_ and AUC_last_ (Figure 2).

On the 14th and 1st day after F3 injection, the ratios of *C*_max_ to AUC_last_ were 0.892 and 0.672 in male rats, respectively, whereas female rats had ratios of 1.938 and 1.489, respectively. The results indicated that *C*_max_ and AUC_last_ in male rats remained relatively stable following repeated doses, whereas the values increased significantly in female rats, implying that frequent administrations could improve the bioavailability of F3 in female rats (i.e., the extent to which the drug was absorbed and utilized in rats). After repeated subcutaneous injection, F3 showed a lower apparent half-life of elimination and a shorter circulation time, suggesting that there is no F3 accumulation in the plasma (Table 3).

### 3.2. Tissue Distribution of F3

Following that, we studied the tissue distribution of F3 after a single subcutaneous injection at 1.0 mg/kg. The presence of F3 in plasma samples and tissue samples was detected using the LC-MS/MS analysis. F3 was only found in plasma samples collected at 1, 2, and 4 hours after the injection. No detectable F3 was found in plasma samples collected at other time points or in any tissue samples (tissue categories include the heart, brain, liver, kidney, lung, spleen, stomach, small intestine, testis, ovaries, subcutaneous fat, and skeletal muscle), suggesting that the concentration was below the lower limit of quantification (LOQ in tissues was 40–80 ng/g).

F3 concentrations in plasma samples peaked 1 hour after administration at 165.18 ± 46.54 ng/mL in males and 174.30 ± 42.96 ng/mL in females and subsequently steadily declined. After 2 hours, the mean plasma F3 concentration was 104.33 ± 25.52 ng/mL in males and 49.68 ± 1.71 ng/mL in females. At the 4th hr, the plasma concentrations dropped to 20.00 ± 9.73 ng/mL in male rats and to 19.42 ± 5.96 ng/mL in female rats.

### 3.3. Acute Toxicity Analysis

The acute toxicity of F3 was then examined with single and repeated subcutaneous injections. PBS, a single dose of F3 (1.0, 10.0, and 100 mg/kg, respectively), or repeated doses of F3 for 14 days (10.0 mg/kg, once a day) were randomly administered subcutaneously to rats. As described above, plasma and tissue samples were collected fifteen days after the first dose of F3.

#### 3.3.1. Food Intake and Bodyweight Gain

**Figure 3:**
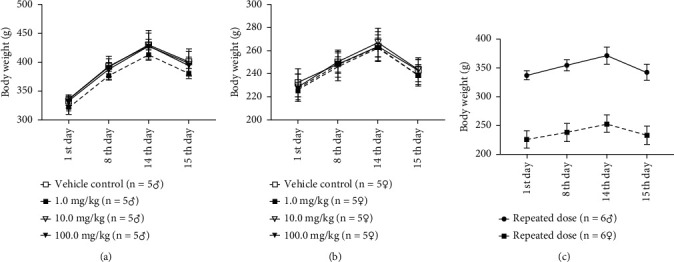
Mean and standard deviation of body weight over time in acute toxicology experiment of male and female SD rats with single or repeated injection. (a) and (b) are the body weight changes of male and female rats with single subcutaneous injection of different concentrations of F3. (c) shows the body weight changes of rats after 14 consecutive days of subcutaneous injection of F3. The body weight of rats after an overnight fast was measured on the 15th day. The body weights of male and female rats show a proportionate increase during the repeated acute toxicology experiment.

A single or repeated subcutaneous injection of F3 had no effect on food intake, body weight, and weight change in rats. During repeated dosage, the body weights of both male and female rats increase proportionately.  Figure 3 and Figure 3 show the changing curves of body weight changes in male and female rats that received a single subcutaneous injection of different concentrations of F3. The changes of body weight after 14 days of subcutaneous injection of F3 are shown in Figure 3. On the 15th day, rats' body weight was also measured after an overnight fast. There were no significant differences in body weight or food intake among the experimental groups and the control group (Figure 3).

#### 3.3.2. Functional Observational Battery

In any of the cases, no rats receiving F3 were found dead or dying by cage observation, and all rats were in a healthy mental state with actively free moments. The changes in the rats' fur, eyes, and mucosa, along with their respiratory system, circulatory system, nervous system, as well as the behaviors were meticulously documented. All subjects showed no abnormalities after receiving a single subcutaneous injection of F3 at 1.0 mg/kg or 10.0 mg/kg. However, one male and two female rats on day 8 and two female rats on day 15 developed dorsal scabs after receiving a single dose of 100.0 mg/kg. During the repeated-dose acute toxicity investigation, scabs were discovered at the injection site in one female rat, three female rats, and two male rats on the 8th, 12th, and 15th days, respectively. These phenomena were considered to be related to F3 administration.

#### 3.3.3. Clinical Biochemistry Analysis

**Figure 4:**
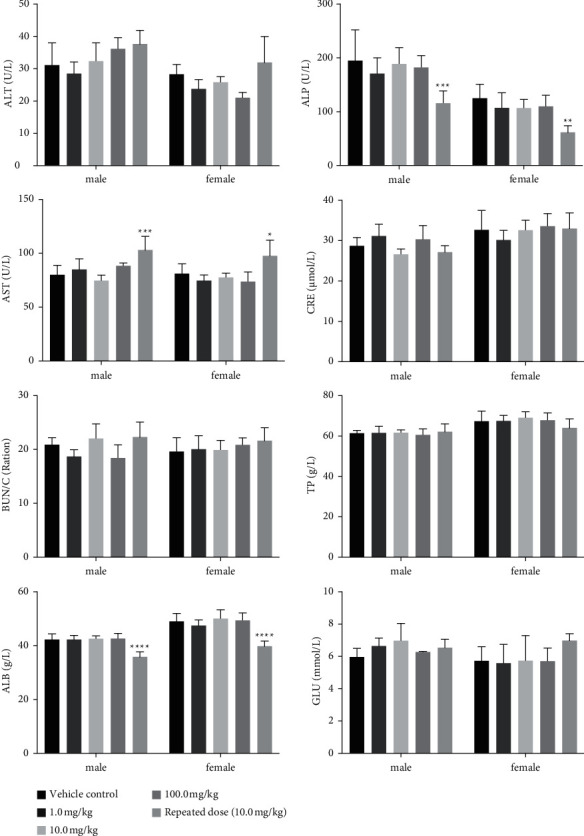
Liver and kidney functions and other important indexes of rats. Rats were treated with 0.9% sodium chloride injection, a single dose of F3 at 1.0 mg/kg, 10.0 mg/kg, or 100.0 mg/kg, and repeated doses of F3 at 10.0 mg/kg for 14 consecutive days. In comparison to the control group, ALP and ALB were decreased, while ALP was increased, with statistical significance. ^*∗*^Significantly different at *P* < 0.05 with vehicle control group.

Biochemical examination on day 15 after F3 single-dose administration revealed no abnormal parameters in male or female rats that received a single injection of different doses of F3 (Tables S1 and S2), including hepatic and renal functions as well as other important parameters (Figure 4). In comparison to the control group, female rats that were given 1.0 mg/kg or 100.0 mg/kg of F3 had significantly lower levels of alanine aminotransferase (ALT) in their blood. However, because this difference was only found in one sex and there was no evidence of a dose-effect relationship of F3, the reduction in ALT level was not considered to be produced directly by F3 treatment.

In rats given 10.0 mg/kg of F3 once per day for 14 consecutive days, no abnormal blood biomedical parameter values were found, indicating that the body function was maintained in a normal physiological condition (Tables S1 and S2). Meanwhile, significant differences in several blood indexes were observed when comparing the values of the experimental rats to those of the control rats: the rats that received repeated dosages exhibited lower ALP and ALB and higher ALT. The increase of lactate dehydrogenase (LDH) in some male rats was considered an occasional phenomenon unrelated to F3. Other blood biochemical indicators in the blood were within the normal limits.

#### 3.3.4. Hematological Analysis

The hematological and coagulation indexes of rats were unaffected by single subcutaneous injections of different doses of F3 (1.0, 10.0, and 100 mg/kg, respectively) or repeated injections of F3 at 10.0 mg/kg for 14 days (Tables 4 and 5).

#### 3.3.5. Gross Necropsy and Weight of Vital Organs

Neither single subcutaneous injection of different doses of F3 (1.0, 10.0, and 100.0 mg/kg) nor 14-day subcutaneous injections of F3 at 10.0 mg/kg affect the weight of the organs, the ratio of viscera to the body, and the ratio of viscera to the brain (Tables 6 and 7).

Gross necropsy showed that the subcutaneous injection of F3 at 100.0 mg/kg caused skin scab and thickening at the administration site in all male rats and four female rats. One male rat, in particular, showed scattered bleeding at the injection site, which was thought to be related to the local administration of high levels of F3.

All rats who received F3 at 10.0 mg/kg for 14 days experienced diffuse subcutaneous bleeding at the injection site. In addition, thickening skin was observed in all males and four females, with scabby skin in one male and three females. The accumulation of F3 local dosage was considered to be the cause of the aforementioned events.

#### 3.3.6. Histopathologic Examinations of Vital Organs

**Figure 5:**
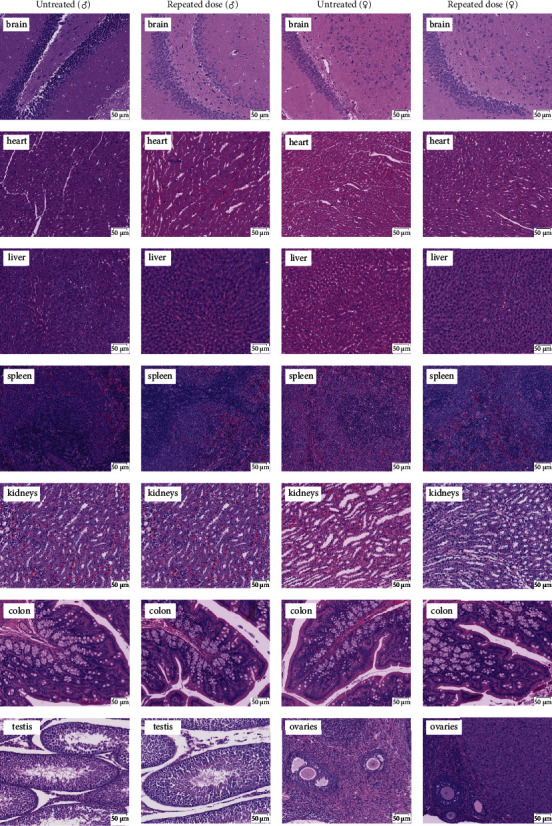
Representative photomicrographies of tissues of rats from control group and repeated dose of F3 groups.

There were no apparent abnormalities, such as excessive hematopoietic cell infiltration, hemorrhage, or structural changes, in essential organs from rats treated with F3 for 14 days at 10.0 mg/kg under histopathologic examinations (Figure 5).

## 4. Discussion

In this study, we found that subcutaneous injection of Caerin 1.9 was well tolerated in SD rats. No accumulation of Caerin 1.9 in plasma was observed after 14 days of repeated subcutaneous injection of 10.0 mg/kg. Except in the peripheral blood, Caerin 1.9 was barely detectable in essential organs and tissues.

No dead or dying rats were observed following a single or repeated *s.c.* injections of F3, even when the single dose was up to 100 mg/kg. F3 had no influence on absolute and relative organ weight, as well as gross and histopathological changes in the heart, brain, spleen, liver, kidney, colon, testis, and ovary, showing that F3 did not induce any significant abnormalities. H&E staining of the heart, liver, kidney, and other important organs remained in normal morphology (Figure 5), indicating that no structural changes and no hematopoietic cell infiltration have occurred. Slight weight changes in several organs, such as the liver and kidney, were not considered treatment-relevant because neither gender showed such a similar appearance. Other studies have indicated that minor changes in organ weight were insufficient to support the conclusion that the candidate treatment has a direct effect on organ weight in rats [27]. The total body weights of male and female rats in the experimental groups were similar to those of control rats (Figure 3). However, due to the short observation period, chronic toxicological studies of F3 are required to explore whether it has a long-term toxic effect on different organs.

Blood has long been used as a marker of pathological and physiological status in humans and animals which is well documented [27]. In acute and chronic toxicological studies, changes in hematological indexes and biochemical parameters are commonly used as indicators of toxicities. In this study, the liver and kidney functions in rats were nearly unaffected compared to control rats (Figure 4). In contrast to the control group, repeated treatment resulted in lower levels of ALP and ALB and higher levels of ALT. The mechanism of changes remains unclear and will require further research and prolonged observation.

Scab was observed at the injection site in rats given a single 100.0 mg/kg subcutaneous injection or repeated dose of 10.0 mg/kg but not in the control rats or rats receiving a single 1.0 mg/kg or 10.0 mg/kg injection [28]. The 14-day 10.0 mg/kg treatment group had a higher incidence of scab development compared to the single 100.0 mg/kg treatment group. The formation of the scab was most likely caused by excessive local F3 concentration and/or the accumulation of F3 in the skin after multiple injections. The results of ABNEUT and ABMONO in the 14-day 10.0 mg/kg treatment group were slightly higher than those in the single-dose groups, which could be due to an increase in inflammatory response after repeated injection [29]. In the follow-up experiment, we will look at the changes in the local injection site to make a more informed decision. In general, the scab was minor, with no obvious consequences on normal activities and behavioral changes in rats, indicating that the wound is of low severity [9, 30, 31]. Overall, the results suggested that F3 was safe at the dosages used in the study.

The pharmacokinetics of subcutaneous injection of F3 were investigated. *T*_max_ was between half an hour and one hour, showing that F3 was well absorbed and had a quick onset (Figure 2 and Table 3). On the other hand, Caerin 1.9 had a short serum half-life of 0.677–4.58 h, which might limit its therapeutic potential in bodies. Modifying the drugs dose has been demonstrated in several studies to increase the serum half-life of drugs. For example, DARPin-MMAF is a targeted drug for the treatment of colon cancer whose serum half-life can be prolonged from 11 minutes to 20.6 hours by conjugating with the unstructured polypeptides PAS or XTEN at different lengths [32]. The half-life of Caerin 1.9 in peripheral circulation can be extended by modifying specific amino acids to achieve a greater therapeutic benefit. As the drug concentration was increased, *T*_1/2_ was prolonged, followed by increases in *C*_max_, AUC_0−last_, and steady-state volume of distribution. Future research could focus on adjusting F3 concentration to obtain optimal bioavailability.

Male and female rats had distinct pharmacokinetic characteristics for Caerin 1.9. This situation is not unique. In animals, gender differences in pharmacokinetics and pharmacodynamics are common [33]. *C*_max_ and AUC_last_ in male rats were higher than those in female rats at first dosage, while, after 14 days of injections, the values of *C*_max_ and AUC_last_ between male and female rats were closed. *C*_max_ and AUC_last_ in male rats remained at a similar level after one dose or multiple doses of F3, whereas female rats had a significantly higher *C*_max_ and AUC_last_ after 14 doses. These showed that repeated administration of Caerin 1.9 increased the serum concentration exclusively in female rats. The underlying mechanism of male-female differences is unknown. Caerin 1.9 possessed a short circulation time in peripheral blood and a short half-life of elimination, as evidenced by the lack of accumulation in the plasma after repeated doses. Similarly, a study of fucoidan, a fucose-rich polysaccharide from brown algae which has been used in transdermal formulations targeting inflammatory skin conditions for the treatment of thrombosis, vascular permeability diseases, subcutaneous wounds, and burns, found no accumulation of the drugs in the plasma after daily dosing with 100 mg/kg for five days [26].

The presence of Caerin 1.9 was barely detectable in all investigated tissues and organs after subcutaneous injection in 24 h, which was similar to the prior findings in the xenograft breast cancer model. According to biodistribution data, the highest uptake of ^125^I labeled F3 in the liver, spleen, kidney, lung, and tumor appeared at 1 hour after *i.v.* injection, but only trace amounts could be detected after 24 hours [19]. The absence of F3 in tissues and organs in this study is thought to be due to the detection time and method used. Furthermore, because Caerin 1.9 is a polypeptide, it may be swiftly degraded by a protease that is not present in plasma. In the future, we plan to investigate the tissue distribution of F3 by combining it with protease inhibitors and absorption enhancers or performing chemical modifications to reduce the effect of proteolysis *in vivo* [34, 35].

In our recent study, intraperitoneal injections of HPV16 E7 peptides, anti-PD-1, and anti-IL-10R antibody, combined with intratumoral injections of Caerin 1.1 and Caerin 1.9 in subcutaneous tumor-bearing mice, significantly reduced tumor growth rate and prolonged the survival time of tumor-bearing mice [14]. As a result, the Caerin peptide treatment was highly effective in boosting the efficacy of immunotherapy by modifying the tumor suppressive microenvironment, and Caerin 1.1 and Caerin 1.9 have pronounced antitumor effects *in vivo* and are worthy of further development as antitumor medications in combination with cancer immunotherapeutic reagents in clinical practice.

In conclusion, Caerin 1.9 (F3) is safe, does not cause death in rats, and has no significant side effects on organ weight, hematological and biochemical parameters, and no significant abnormalities in the gross and histopathological appearance of major organs, with the exception of skin scab induced by a high dose or repeated injections. Caerin 1.9 could be an ideal candidate for combination with current therapies for better management of solid tumor [36].

## Figures and Tables

**Table 1:** Tissue samples collected for biodistribution analysis.

Organ	Collection site	Organ	Collection site
Heart	Left ventricular myocardium^a^	Testis	Right and left upper testicles
Brain	Brain^b^	Ovary	Left and right ovaries
Liver	Upper left lobe of liver	Subcutaneous fat	Inguinal region
Kidney	Upper part of left and right kidney	Stomach	Close to cardia
Lungs	Left and right lung root	Skeletal muscle	Dorsal muscle
Spleen	Upper part of spleen	Small intestine	Duodenum^c^

^a^Near the atrioventricular orifice. ^b^Consists of cortex and medulla, mixed together and homogenized. ^c^Removal of intestinal contents.

**Table 2:** Experimental group design.

Test substance	Dose , (mg/kg)	Volume (ml/kg)	Concentration (mg/ml)	Number of animals(M/F)
0.9% NS	0	5	0	5/5
	1.0	5	0.2	5/5

F3	10.0	5	2.0	5/5
	100.0	5	20.0	5/5

M:male; F:female; NS:normal saline.

**Table 3:** Pharmacokinetic parameters of F3 after single and repeated dose administration to rats calculated by WinNonlin software.

Pharmacokinetic parameters	Single dose (1.0 mg/kg)		Repeated dose (10.0 mg/kg)			
			1st dose		14th dose	
	Male	Female	Male	Female	Male	Female
*T*_1/2_ (h)	1.16	0.677	4.58	1.33	0.894	1.03
*T*_max_ (h)	1.00	0.500	1.00	1.00	0.500	1.00
*C*_max_ (ng/mL)	256	110	591	256	527	496
AUC_0−last_ (h × ng/mL)	661	216	1890	740	1270	1102
AUC_0−Inf_ (h × ng/mL)	686	222	1906	751	1274	1110
AUC__%Extrap__obs (%)	3.62	2.88	0.807	1.48	0.365	0.658
MRT_Inf__obs (h)	2.05	1.42	3.26	2.18	2.06	1.91
Vz_F_obs (mL/kg)	2431.95	4402.21	34647.55	25498.49	10121.99	13419.28
Cl_F_obs (mL/h/kg)	1458.26	4504.48	5247.61	13313.00	7846.61	9011.18
AUC_last_/D (h × mg/mL)	661	216	189	74	127	110
C_max_ ratio of M/F	2.327		2.309		1.063	
AUC_last_ ratio of M/F	3.060		2.554		1.152	
14th/1st ratio of *C*_max_	——		Male: 0.892, female: 1.938			
14th/1st ratio of AUC_last_	——		Male: 0.672, female: 1.489			

**Table 4:** Hematological values of male rats.

Parameters	Single dose				Repeated dose
	Vehicle control	1.0 mg/kg	10.0 mg/kg	100.0 mg/kg	10.0 mg/kg
RBC (10^12^/L)	7.838 ± 0.324	8.163 ± 0.679	7.916 ± 0.383	7.742 ± 0.232	6.94 ± 0.18
HCT (%)	47.7 ± 1.72	48.4 ± 1.29	47.5 ± 1.89	47.62 ± 1.59	42.18 ± 0.92
HGB (g/dL)	15.08 ± 0.49	15.1 ± 0.41	14.72 ± 0.59	14.6 ± 0.55	12.68 ± 0.34
MCV (fL)	60.88 ± 1.65	59.58 ± 4.36	60.06 ± 2.02	61.52 ± 0.65	60.78 ± 1.44
MCH (pg)	19.24 ± 0.43	18.63 ± 1.63	18.6 ± 0.49	18.86 ± 0.15	18.28 ± 0.56
MCHC (g/dL)	31.62 ± 0.38	31.23 ± 0.69	30.98 ± 0.24	30.66^*∗∗*^ ± 0.36	30.08 ± 0.52
ABRETIC (10^9^/L)	257.42 ± 45.92	250.78 ± 19.32	252.72 ± 25.77	270.82 ± 25.29	338.6 ± 20.2
WBC (10^9^/L)	10.148 ± 1.419	9.315 ± 0.344	12.12 ± 2.998	10.654 ± 1.291	13.19 ± 3.49
ABNEUT (10^9^/L)	1.412 ± 0.348	1.185 ± 0.375	1.498 ± 0.573	1.742 ± 0.58	2.43 ± 1.39
ABLYMP (10^9^/L)	8.378 ± 1.263	7.695 ± 0.12	10.178 ± 2.676	8.444 ± 1.168	10.1 ± 2.26
ABMONO (10^9^/L)	0.224 ± 0.083	0.283 ± 0.124	0.262 ± 0.043	0.27 ± 0.06	0.34 ± 0.09
ABBASO (10^9^/L)	0.008 ± 0.004	0.005 ± 0.006	0.012 ± 0.008	0.01 ± 0.01	0.01 ± 0.01
ABEOS (10^9^/L)	0.07 ± 0.043	0.088 ± 0.013	0.086 ± 0.059	0.11 ± 0.062	0.17 ± 0.06
PLT (10^9^/L)	862.8 ± 111.4	862.8 ± 95.1	878 ± 118.5	985.4 ± 65	1421.5 ± 225.3
MPV (fL)	9.06 ± 0.19	9.35 ± 0.33	9.62^*∗*^ ± 0.41	9.26 ± 0.24	9.05 ± 0.36
APTT (seconds)	17.46 ± 1.32	17.44 ± 0.85	17.86 ± 1.84	17.34 ± 1.96	16.2 ± 1.82
PT (seconds)	20.18 ± 1.22	19.76 ± 0.92	19.88 ± 0.94	20.14 ± 0.95	20.87 ± 1.2

Values are expressed as mean ± SD.

**Table 5:** Hematological values of female rats.

Parameters	Single dose				Repeated dose
	Vehicle control	1.0 mg/kg	10.0 mg/kg	100.0 mg/kg	10.0 mg/kg
RBC (10^12^/L)	7.778 ± 0.219	7.584 ± 0.568	7.954 ± 0.424	8.042 ± 0.561	6.04 ± 0.37
HCT (%)	46.06 ± 0.6	45.34 ± 3.13	46.98 ± 1.22	47.16 ± 1.49	38.2 ± 1.34
HGB (g/dL)	14.92 ± 0.23	14.44 ± 1.06	14.9 ± 0.29	14.96 ± 0.47	11.62 ± 0.50
MCV (fL)	59.26 ± 1.3	59.86 ± 0.56	59.1 ± 1.71	58.8 ± 2.76	63.38 ± 2.38
MCH (pg)	19.2 ± 0.58	19.06 ± 0.27	18.78 ± 0.75	18.64 ± 1	19.25 ± 0.69
MCHC (g/dL)	32.38 ± 0.3	31.82 ± 0.47	31.76 ± 0.46	31.68^*∗*^ ± 0.33	30.4 ± 0.46
ABRETIC (10^9^/L)	191.58 ± 55.04	226.5 ± 33.41	192.02 ± 21.07	215.84 ± 21.41	273.87 ± 31.75
WBC (10^9^/L)	7.444 ± 1.547	8.022 ± 3.840	7.922 ± 1.865	7.28 ± 2.163	10.67 ± 1.900
ABNEUT (10^9^/L)	1.146 ± 0.512	1.71 ± 1.516	1.128 ± 0.308	1.148 ± 0.434	2.53 ± 0.45
ABLYMP (10^9^/L)	5.988 ± 1.053	5.956 ± 2.336	6.526 ± 1.92	5.886 ± 1.807	7.64 ± 1.61
ABMONO (10^9^/L)	0.18 ± 0.08	0.228 ± 0.234	0.136 ± 0.034	0.142 ± 0.094	0.23 ± 0.08
ABBASO (10^9^/L)	0.006 ± 0.005	0.006 ± 0.005	0.01 ± 0.007	0.008 ± 0.004	0.01 ± 0
ABEOS (10^9^/L)	0.066 ± 0.011	0.072 ± 0.034	0.072 ± 0.032	0.06 ± 0.031	0.16 ± 0.07
PLT (10^9^/L)	979.8 ± 82.6	1081.6 ± 83.2	1004.4 ± 93.7	987 ± 86.1	1357.67 ± 169.65
MPV (fL)	8.92 ± 0.25	9.02 ± 0.30	9.28 ± 0.20	9 ± 0.19	8.58 ± 0.42
APTT (seconds)	16.8 ± 0.65	16.94 ± 1.15	18.24 ± 1.36	17.02 ± 1.47	14.9 ± 0.28
PT (seconds)	20.04 ± 0.91	20.06 ± 0.75	19.44 ± 0.70	19.72 ± 0.50	20.45 ± 1.16

Values are expressed as mean ± SD.

**Table 6:** Absolute and relative weights of organs of male rats.

Parameters	Single dose				Repeated dose
	Vehicle control	1.0 mg/kg	10.0 mg/kg	100.0 mg/kg	10.0 mg/kg
Body weight (g)	400.42 ± 22.87	380.74 ± 8.63	397.34 ± 21.46	394.58 ± 14.44	342.57 ± 13.86
Brain (g)	2.1834 ± 0.0884	2.163 ± 0.0999	2.181 ± 0.1198	2.1528 ± 0.0816	2.062 ± 0.1162
Heart (g)	1.7608 ± 0.3317	1.485 ± 0.1408	1.728 ± 0.2013	1.6352 ± 0.0401	1.5727 ± 0.082
Liver (g)	12.7004 ± 1.7057	11.4326 ± 0.5122	12.5468 ± 0.8106	12.2242 ± 0.7073	9.9218 ± 0.6297
Spleen (g)	0.8548 ± 0.214	0.7378 ± 0.14	0.8204 ± 0.1012	0.793 ± 0.109	0.741 ± 0.1802
Kidneys (g)	2.988 ± 0.2071	2.8174 ± 0.3469	3.151 ± 0.1131	2.9858 ± 0.1294	2.5043 ± 0.0600
Adrenals (g)	0.059 ± 0.0075	0.0564 ± 0.0078	0.0654 ± 0.0073	0.066 ± 0.0075	0.0612 ± 0.0041
Thymus (g)	0.6074 ± 0.0651	0.545 ± 0.1054	0.6256 ± 0.1022	0.6608 ± 0.082	0.4433 ± 0.0724
Thyroid (g)	0.0478 ± 0.0073	0.0494 ± 0.0051	0.0498 ± 0.0093	0.0482 ± 0.0075	0.0358 ± 0.0092
Testis (g)	3.5324 ± 0.3453	3.328 ± 0.2025	3.2666 ± 0.0813	3.4182 ± 0.3029	3.3857 ± 0.1596
Epididymides (g)	0.9294 ± 0.1662	0.9464 ± 0.0677	0.9564 ± 0.0348	0.9956 ± 0.0459	1.0408 ± 0.0584

*Organ-to-body weight ratio (%)*					
Brain	0.5462 ± 0.0266	0.5684 ± 0.0246	0.5502 ± 0.0414	0.5462 ± 0.0313	0.6027 ± 0.0396
Heart	0.44 ± 0.0831	0.3896 ± 0.0287	0.4362 ± 0.0588	0.4148 ± 0.0148	0.4593 ± 0.0262
Liver	3.161 ± 0.2538	3.0038 ± 0.1462	3.1566 ± 0.0559	3.0962 ± 0.0771	2.8948 ± 0.0939
Spleen	0.212 ± 0.0439	0.1934 ± 0.033	0.2074 ± 0.033	0.201 ± 0.0269	0.2148 ± 0.0437
Kidneys	0.7466 ± 0.0439	0.7386 ± 0.0732	0.794 ± 0.0359	0.757 ± 0.0276	0.7318 ± 0.0218
Adrenals	0.0148 ± 0.0011	0.0148 ± 0.0016	0.0162 ± 0.0018	0.0166 ± 0.0023	0.0178 ± 0.0013
Thymus	0.1526 ± 0.0221	0.1432 ± 0.0257	0.158 ± 0.0301	0.1674 ± 0.0172	0.1292 ± 0.018
Thyroid	0.012 ± 0.0014	0.013 ± 0.0016	0.0126 ± 0.0027	0.012 ± 0.002	0.0105 ± 0.0021
Testis	0.8814 ± 0.0541	0.8742 ± 0.051	0.824 ± 0.0519	0.8678 ± 0.0894	0.9903 ± 0.0744
Epididymides	0.231 ± 0.0319	0.2488 ± 0.0201	0.2412 ± 0.015	0.253 ± 0.0201	0.3047 ± 0.0274

*Organ-to-brain weight ratio (g/g)*					
Heart	80.392 ± 13.13	68.668 ± 5.823	79.07 ± 5.614	76.094 ± 4.612	76.512 ± 6.581
Liver	581.294 ± 71.985	530.106 ± 45.202	576.69 ± 48.769	568.862 ± 45.911	482.405 ± 39.55
Spleen	38.948 ± 8.538	34.02 ± 5.771	37.666 ± 4.57	36.82 ± 4.84	35.907 ± 8.243
Kidneys	136.806 ± 6.963	130.33 ± 15.192	144.666 ± 5.6	138.964 ± 10.232	121.752 ± 6.906
Adrenals	2.704 ± 0.368	2.606 ± 0.333	2.994 ± 0.233	3.066 ± 0.335	2.978 ± 0.32
Thymus	27.848 ± 3.183	25.276 ± 5.165	28.58 ± 3.285	30.734 ± 4.031	21.58 ± 3.924
Thyroid	2.192 ± 0.351	2.292 ± 0.316	2.286 ± 0.415	2.238 ± 0.322	1.737 ± 0.425
Testis	161.986 ± 17.165	153.876 ± 6.445	150.056 ± 7.131	158.602 ± 9.431	164.705 ± 13.717
Epididymides	42.52 ± 7.181	43.792 ± 3.12	43.986 ± 3.405	46.262 ± 1.845	50.623 ± 4.213

Values are expressed as mean ± SD.

**Table 7:** Absolute and relative weights of organs of female rats.

Parameters	Single dose				Repeated dose
	Vehicle control	1.0 mg/kg	10.0 mg/kg	100.0 mg/kg	10.0 mg/kg
Body weight (g)	242.98 ± 9.59	238.86 ± 7.94	243.48 ± 10.53	238.86 ± 9.44	233.55 ± 16.03
Brain (g)	2.0674 ± 0.0674	2.0504 ± 0.0552	2.0232 ± 0.044	1.9918 ± 0.0665	1.9517 ± 0.0244
Heart (g)	1.0142 ± 0.104	1.1086 ± 0.1559	0.999 ± 0.0943	1.0914 ± 0.0821	1.1247 ± 0.1287
Liver (g)	7.7002 ± 0.7548	7.7464 ± 0.2893	7.3692 ± 0.709	7.8582 ± 0.5028	7.5278 ± 0.6229
Spleen (g)	0.5588 ± 0.0540	0.57 ± 0.1214	0.5508 ± 0.0854	0.545 ± 0.0571	0.6908 ± 0.0750
Kidneys (g)	1.9894 ± 0.1988	1.9364 ± 0.118	1.8596 ± 0.1438	1.924 ± 0.0685	1.7017 ± 0.123
Adrenals (g)	0.0748 ± 0.0118	0.0728 ± 0.0097	0.0758 ± 0.0114	0.076 ± 0.0116	0.0793 ± 0.0101
Thymus (g)	0.5176 ± 0.0353	0.5216 ± 0.0746	0.535 ± 0.0762	0.478 ± 0.0725	0.427 ± 0.0886
Thyroid (g)	0.0308 ± 0.0039	0.0282 ± 0.0029	0.0338 ± 0.005	0.0344 ± 0.0057	0.0242 ± 0.0053
Uterus (g)	0.5624 ± 0.1174	0.6212 ± 0.0948	0.4664 ± 0.1167	0.5842 ± 0.0922	0.4797 ± 0.1336
Ovaries (g)	0.1002 ± 0.0182	0.1036 ± 0.0242	0.096 ± 0.0182	0.108 ± 0.0223	0.093 ± 0.0151

*Organ-to-body weight ratio (%)*					
Brain	0.8514 ± 0.0271	0.8588 ± 0.0192	0.8318 ± 0.0335	0.8342 ± 0.0162	0.8388 ± 0.0590
Heart	0.4168 ± 0.0364	0.4632 ± 0.0555	0.4104 ± 0.0365	0.4576 ± 0.0363	0.4815 ± 0.0436
Liver	3.1644 ± 0.2149	3.2444 ± 0.1224	3.0258 ± 0.2399	3.29 ± 0.1695	3.2212 ± 0.0775
Spleen	0.2298 ± 0.0182	0.2382 ± 0.0477	0.2252 ± 0.0267	0.2278 ± 0.018	0.2968 ± 0.036
Kidneys	0.8182 ± 0.0641	0.8106 ± 0.0353	0.764 ± 0.0516	0.806 ± 0.029	0.7308 ± 0.0653
Adrenals	0.0308 ± 0.004	0.0304 ± 0.005	0.031 ± 0.0042	0.0316 ± 0.0043	0.034 ± 0.0034
Thymus	0.2134 ± 0.0167	0.2184 ± 0.0331	0.2192 ± 0.0247	0.2012 ± 0.0371	0.1817 ± 0.0285
Thyroid	0.0126 ± 0.0017	0.0118 ± 0.0008	0.0138 ± 0.0016	0.0144 ± 0.0027	0.0105 ± 0.0023
Uterus	0.2308 ± 0.0433	0.2602 ± 0.0375	0.1914 ± 0.0455	0.2442 ± 0.0337	0.2067 ± 0.0629
Ovaries	0.0412 ± 0.0066	0.0432 ± 0.0094	0.0394 ± 0.0067	0.0452 ± 0.0084	0.0397 ± 0.0044

*Organ-to-brain weight ratio (g/g)*					
Heart	49.016 ± 4.291	53.986 ± 6.753	49.414 ± 4.975	54.812 ± 3.894	57.62 ± 6.476
Liver	372.406 ± 34.108	378.002 ± 17.196	364.644 ± 39.044	394.656 ± 24.796	385.71 ± 31.435
Spleen	27.062 ± 2.869	27.816 ± 5.976	27.206 ± 4.067	27.338 ± 2.415	35.388 ± 3.677
Kidneys	96.252 ± 9.422	94.388 ± 3.839	91.964 ± 7.656	96.64 ± 3.443	87.178 ± 6.079
Adrenals	3.62 ± 0.579	3.556 ± 0.528	3.748 ± 0.558	3.814 ± 0.545	4.06 ± 0.477
Thymus	25.066 ± 1.98	25.46 ± 3.812	26.42 ± 3.537	24.094 ± 4.264	21.867 ± 4.445
Thyroid	1.492 ± 0.194	1.374 ± 0.111	1.67 ± 0.243	1.732 ± 0.323	1.238 ± 0.267
Uterus	27.082 ± 4.803	30.302 ± 4.505	23.074 ± 5.887	29.296 ± 4.16	24.592 ± 6.939
Ovaries	4.836 ± 0.799	5.052 ± 1.175	4.75 ± 0.919	5.412 ± 1.018	4.763 ± 0.76

Values are expressed as mean ± SD.

## Data Availability

The data used to support the findings of this study are included within the article and Supplementary Materials.

## Supplementary Materials

Supplementary Materials Supplementary Materials are the method validation data for the LC-MS/MS from pharmacokinetic analysis and tissue distribution of F3 and serum biochemical data of rats from acute toxicological study of single and repeated subcutaneous injection of F3.
