# Supplementary material for: Preclinical Pharmacokinetics, Biodistribution, and Acute Toxicity Evaluation of Caerin 1.9 Peptide in Sprague Dawley Rats
Source: Evid Based Complement Alternat Med. 2022 Aug 2;2022:9869293. doi: 10.1155/2022/9869293 (PMC9363195; doi:10.1155/2022/9869293)
Supplement: Supplementary Materials — are the method validation data for the LC-MS/MS from pharmacokinetic analysis and tissue distribution of F3 and serum biochemical data of rats from acute toxicological study of single and repeated subcutaneous injection of F3. [file 9869293.f1.docx]

**Preclinical pharmacokinetics, biodistribution and acute toxicity evaluation of caerin 1.9 peptide in Sprague Dawley rats**

**1.Method Validation**

**1.1Sensitivity**

Representative chromatograms of F3 are shown in below, including a blank plasma sample **(Fig. S1A)** and a blank plasma sample spiked with F3 at 2 ng/mL **(Fig. S1B)**. No interfering peaks were observed at the retention times of F3 in the blank sample. The response intensity of F3 was sufficient for quantification at the concentration of 2 ng/ mL (LLOQ).

**Fig. S1A**

**Fig. S1B**

**Fig. S1.Representative chromatograms of F3**

**1.2 Carry-Over**

A blank sample was analyzed following the ULOQ to evaluate carryover. No peak was found at the retention time of F3 in the blank sample, indicating that

the carryover of the method was acceptable.

**1.3 Calibration Curve and Linearity**

The plasma calibration curve was constructed by eight calibrators over the concentration range of 2~1000 (2, 5, 10, 20, 50, 100, 500 and 1000 ng/mL) in duplicate were used to construct calibration curves for F3 in male SD rat plasma. The calibration curves were fitted using a least square linear regression model y=2.05e-006x2+0.00747x-0.000230, weighted by 1/x^2^ using Analyst software **(Fig. S2)**.


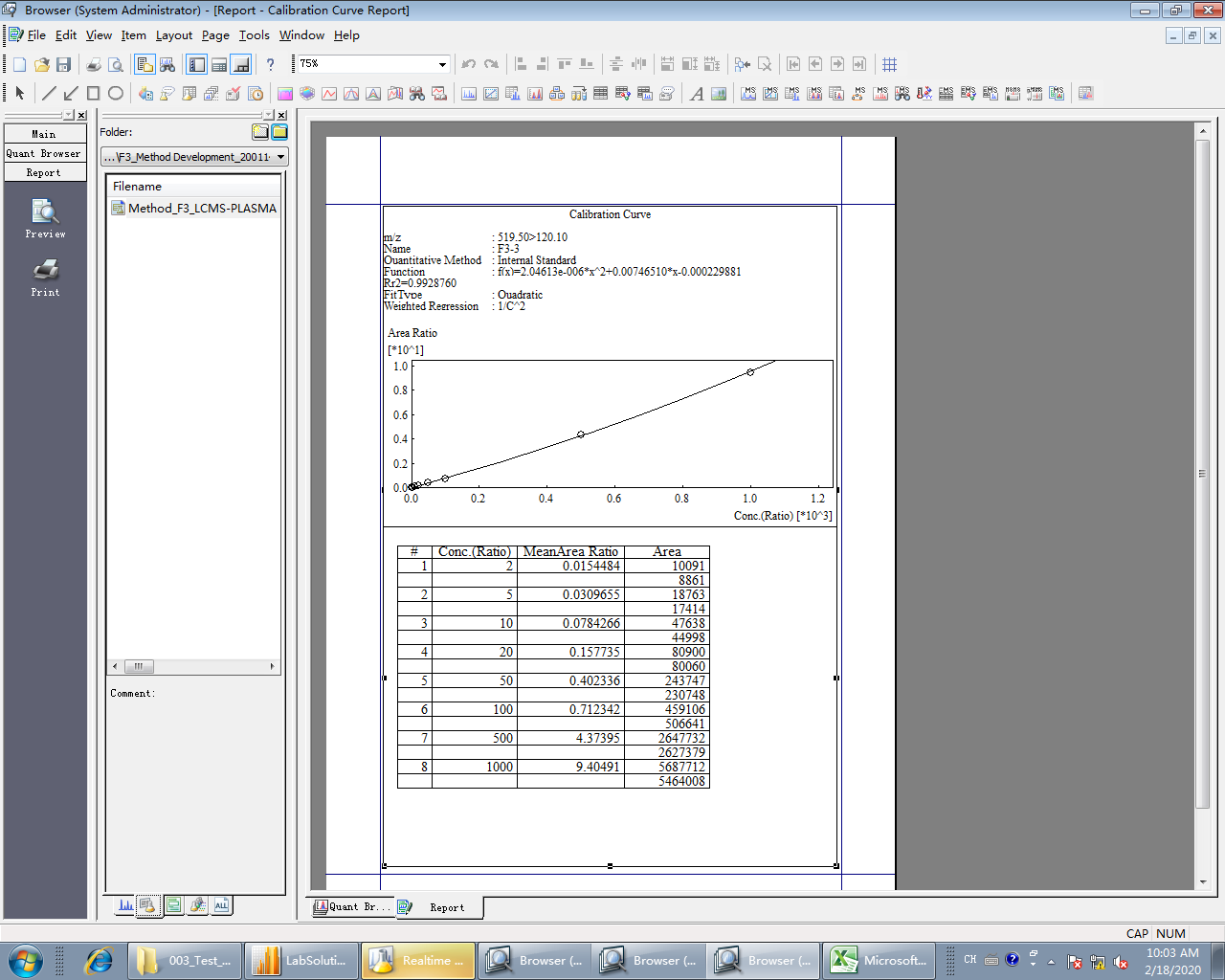


**Fig. S2. Calibration Curve and Linearity**

**1.4 Accuracy and Precision**

The intra- precision and accuracy were evaluated by parallel analytical runs performed. Five replicates of F3 at four QC levels (2, 6, 100 and 800 ng/mL) were analyzed.

The intra- precision (% RSD) of F3 was in the range of 6.23 ~ 13.5%. The accuracy ranged from -1.51 to 12.9%.

The intra-batch and inter-batch accuracy of QC samples at five concentration levels met the acceptance criteria, and the results indicate that the method had good accuracy and excellent precision.

**1.5 Matrix Effects**

The matrix of the method was evaluated by comparing the peak areas of F3 at QC levels (800 ng/mL) with three replicates, including standards and standards spiked after extraction. In this study, the matrix values were 99.3±33.1, indicating that no matrix effect for F3 in the method.

**1.6 Extraction Recovery**

The extraction recovery of the method was evaluated by comparing the peak areas of F3 at two QC levels (6 and 800 ng/mL) with three replicates, including standards spiked before extraction and standards spiked after extraction. Under these operating conditions, the detailed values of recoveries in rat plasma were 82.8±8.20% and 77.0±4.40%.

**1.7 Matrix Stability**

Three replicates of F3 at two QC levels (6 and 800 ng/mL) were analyzed. All stability of QC samples for F3 were studied under different possible conditions, which included matrix freeze-thaw stability (three cycles, -75±15℃/22℃), stability in the auto-sampler at 5±3ºC for 24 h, bench-top stability at room temperature for 24 h, and stability for 4 h at room temperature. The detailed values of remaining percentage (%) were 92.3% ~110%.

**1.8 LC-MS Method**

The analyte was assayed by a liquid chromatography coupled with tandem mass spectrometry (LC-MS/MS) method. The LC-MS/MS system consisted of a Shimadzu HPLC system (Kyoto, Japan) and LCMS-8060 instrument (Serial NO. 011105500332 AE). LC conditions were as follows: column, HALO ES-C18 2.7 µ C18 160A (50*2.1 mm); gradient elutuion at 0.5 mL/min with 5 %acetonitrile in water containing 0.1% formic acid or 95% acetonitrile in water containing 0.1% formic acid; and injection volume, 10 µL. A multiple reaction monitoring (MRM) method was selected for quantitative analysis. The optimized transitions were 519.50>129.15 (five charges) and 649.15>120.10 (four charges) and 393.20>373.25, 237.15>194.15 for analyte and internal standard, respectively. The instrumental parameters were set as follows: ion spray voltage: Nebulizing gas flow: 3 L/min; Heating gas flow: 10 L/min; Interface temperature : 300 ℃; DL temperature: 250 ℃; Heat block temperature: 400 °C.

The desired serial concentrations of working solutions were achieved by diluting stock solution (1 mg/mL in DMSO) of analyte with 50% acetonitrile in water. 5 μL of working solutions (10 to 10000 ng/mL) were added to 50 μL of the blank rat plasma and tissues to achieve calibration standards or quality control (QC) samples of 1 to 1000 ng/mL in a total volume of 55 μL.

The resulting standard, QC, or study samples were added to 200 μL of methanol containing IS (50 ng/mL dexamethasone, 2 ng/mL verapamil and 2 ng/mL carbamazepine as internal standard (IS) for protein precipitation. All samples were then vortexed for 30 seconds. After centrifugation at 4°C and 4000 rpm (ca. 3740 x g) for 15 minutes, 100 µL of the supernatant was diluted with 200 µL of water for injection on LC-MS/MS.

PK parameters were estimated by non-compartmental model with the calculation model of "Linear Trapezoidal Linear Interpolation" using WinNonlin 6.1.

**2. Clinical biochemistry analysis**

On day 15 after F3 single-dose and repeated-dose administration, no abnormal parameters were observed by biochemical examination **(Table. S1 and** **Table. S2)**.

**3. Histopathologic examinations of vital organs**

Rats were treated with repeated doses of F3 at 10.0mg/kg for 14 consecutive days. Representative pictures from H&E staining sections of the brain, heart, liver, spleen, colon, testis and ovaries. No significant abnormalities associated with F3 was observed**(Fig. S3)**.

**Table.S1. Serum biochemical data for male rats**

| **Parameters** | Single dose | | | | Repeated dose |
| --- | --- | --- | --- | --- | --- |
|  | Vehicle control | 1.0 mg/kg | 10.0 mg/kg | 100.0 mg/kg | 10.0 mg/kg |
| ALT (U/L) | 31.2±6.8 | 28.8±3.3 | 32.4±5.6 | 36.2±3.5 | 37.67±4.23 |
| AST (U/L) | 80.6±8.4 | 85.2±9.8 | 75.2±4.6 | 88.6±2.3 | 103±13.1 |
| ALP (U/L) | 195.4±56.6 | 170.2±30.5 | 188.8±30.5 | 182.4±22.3 | 115.83±22.95 |
| CK (U/L) | 191±37.1 | 196.6±39 | 178±12.1 | 197±40.6 | 120.83±57.45 |
| LDH (U/L) | 228.42±124.02 | 241.24±75.6 | 151.76±32.34 | 150.26±25.35 | 279.17±103.09 |
| TP (g/L) | 61.22±1.33 | 61.84±3.15 | 61.64±1.6 | 60.68±2.94 | 62.2±3.41 |
| ALB (g/L) | 42.48±1.76 | 42.56±1.37 | 42.76±0.97 | 42.52±2.09 | 35.88±1.8 |
| GLO (g/L) | 18.74±1.68 | 19.28±2.03 | 18.88±1.86 | 18.16±1.85 | 26.32±2.47 |
| A/G (Ratio） | 2.286±0.28 | 2.224±0.189 | 2.284±0.264 | 2.358±0.262 | 1.37±0.12 |
| BU (mmol/L) | 5.308±0.42 | 5.092±0.408 | 5.136±0.564 | 4.894±0.67 | 5.32±0.75 |
| CRE (μmol/L) | 28.8±1.9 | 31.2±2.9 | 26.6±1.3 | 30.4±3.4 | 27.17±1.6 |
| BUN/C (Ratio) | 21.026±1.269 | 18.662±1.414 | 22.058±2.758 | 18.432±2.506 | 22.31±2.8 |
| GLU (mmol/L) | 6.002±0.512 | 6.664±0.478 | 6.994±1.064 | 6.256±0.079 | 6.52±0.54 |
| CHO (mmol/L) | 1.916±0.29 | 1.918±0.237 | 1.89±0.188 | 1.78±0.278 | 1.79±0.13 |
| TG (mmol/L) | 0.442±0.177 | 0.648±0.305 | 0.804±0.393 | 0.708±0.069 | 0.29±0.04 |
| Na (mmol/L) | 150.46±0.6 | 150.42±0.64 | 149.16*±0.78 | 150.38±0.77 | 148.12±1.44 |
| K (mmol/L) | 6.188±0.439 | 6.152±0.603 | 6.294±0.211 | 5.9±0.168 | 7.18±0.7 |
| Cl (mmol/L) | 104.14±1.15 | 104.98±1.38 | 102.76±1.3 | 103.72±1.1 | 105.47±0.98 |
| Ca (mmol/L) | 2.678±0.081 | 2.716±0.058 | 2.742±0.072 | 2.662±0.048 | 2.7±0.04 |
| P (mmol/L) | 3.46±0.18 | 3.76±0.33 | 3.64±0.26 | 3.54±0.21 | 3.65±0.4 |

Values are expressed as Mean ± SD

**Table.S2****. Serum biochemical data for female rats**

| **Parameters** | Single dose | | | | Repeated dose |
| --- | --- | --- | --- | --- | --- |
|  | Vehicle control | 1.0 mg/kg | 10.0 mg/kg | 100.0 mg/kg | 10.0 mg/kg |
| ALT (U/L) | 28.4±2.9 | 23.8*±2.9 | 26±1.6 | 21.2**±1.5 | 32±8 |
| AST (U/L) | 81±9.5 | 75.4±4.6 | 77.8±3.8 | 74.2±8.5 | 97.83±14.3 |
| ALP (U/L) | 125±25.9 | 106.8±28.2 | 107.6±15.2 | 109.8±20.5 | 61.5±12.24 |
| CK (U/L) | 104.8±31.8 | 92.6±7.8 | 104.8±31.6 | 90.2±13 | 113±20.13 |
| LDH (U/L) | 123.72±32.24 | 121.52±29.34 | 135.2±47.94 | 125.92±26.89 | 191.7±39.55 |
| TP (g/L) | 67.46±4.98 | 67.7±2.58 | 69.58±2.15 | 68.08±3.3 | 64.48±4.05 |
| ALB (g/L) | 49.36±2.6 | 47.62±2.05 | 50.34±3.01 | 49.4±2.93 | 39.95±1.80 |
| GLO (g/L) | 18.1±2.77 | 20.08±1.72 | 19.24±1.41 | 18.68±1.46 | 24.53±2.80 |
| A/G (Ratio） | 2.76±0.296 | 2.386±0.244 | 2.636±0.333 | 2.656±0.267 | 1.65±0.180 |
| BU (mmol/L) | 5.674±1.221 | 5.33±0.532 | 5.766±0.655 | 6.212±0.648 | 6.31±0.9 |
| CRE (μmol/L) | 32.8±4.7 | 30.2±2.3 | 32.8±2.2 | 33.8±2.9 | 33.17±3.71 |
| BUN/C (Ratio) | 19.694±2.499 | 20.202±2.412 | 20.024±1.603 | 20.952±1.306 | 21.71±2.25 |
| GLU (mmol/L) | 5.712±0.916 | 5.616±1.125 | 5.742±1.553 | 5.706±0.813 | 7.01±0.400 |
| CHO (mmol/L) | 1.846±0.548 | 1.656±0.355 | 1.548±0.35 | 1.484±0.157 | 1.81±0.23 |
| TG (mmol/L) | 0.178±0.033 | 0.236±0.092 | 0.214±0.042 | 0.18±0.057 | 0.28±0.06 |
| Na (mmol/L) | 148.82±1.55 | 147.9±0.95 | 147.56±1.26 | 148.18±0.91 | 146.6±1.71 |
| K (mmol/L) | 6.356±0.544 | 6.646±0.483 | 6.744±0.595 | 6.686±0.31 | 7±0.66 |
| Cl (mmol/L) | 103.44±0.52 | 104.52±1.49 | 102.82±1.21 | 102.86±1.2 | 104.57±1.26 |
| Ca (mmol/L) | 2.736±0.023 | 2.784±0.106 | 2.778±0.118 | 2.77±0.107 | 2.81±0.07 |
| P (mmol/L) | 3.18±0.13 | 3.56±0.4 | 3.36±0.25 | 3.44±0.22 | 3.25±0.29 |

Values are expressed as Mean ± SD
